# Goldilocks: a tool for identifying genomic regions that are ‘just right’

**DOI:** 10.1093/bioinformatics/btw116

**Published:** 2016-03-07

**Authors:** Samuel M. Nicholls, Amanda Clare, Joshua C. Randall

**Affiliations:** ^1^Department of Computer Science, Aberystwyth University, Aberystwyth, UK; ^2^Department of Human Genetics Informatics, Wellcome Trust Sanger Institute, Cambridge, UK

## Abstract

**Summary**: We present Goldilocks: a Python package providing functionality for collecting summary statistics, identifying shifts in variation, discovering outlier regions and locating and extracting interesting regions from one or more arbitrary genomes for further analysis, for a user-provided definition of interesting.

**Availability and implementation**: Goldilocks is freely available open-source software distributed under the MIT licence. Source code is hosted publicly at https://github.com/SamStudio8/goldilocks and the package may also be installed using pip install goldilocks. Documentation can be found at https://goldilocks.readthedocs.org.

**Contact**: msn@aber.ac.uk

**Supplementary information:** Supplementary data are available at *Bioinformatics* online.

## 1 Introduction

Goldilocks is a Python package designed to take a census of sequence data in order to find regions that meet some criteria that are ‘just right’ for further analysis. It can be used by importing the package into a standalone script or via the command line tool. The tool accepts sequence data which could be from one or more individuals (with one or more sequences from each), organized into chromosomes or contigs and stored as FASTA.

Goldilocks must also be supplied with a desired census strategy defining the criteria of interest, such as occurrences of individual nucleotide bases, motifs, deviations from a reference or GC-content. Chromosomes (or contigs) are slid beneath a window of a user-defined length and overlap and the strategy is applied over all input genomes.

Goldilocks was developed as part of a quality control study to first determine what constitutes ‘bad’ quality data. For a large study, a pipeline was constructed to repeatedly perform variant calling, withholding one sample from the analysis in turn. A given sample’s downstream effect could then be quantified by measuring the difference in the accuracy of a run in the absence of that sample. However, repeated application of computationally expensive and time consuming analysis of whole genomes was unsustainable. The problem space needed to be reduced to a representative region: the genome’s Goldilocks.

For our study, we defined a 1 Mb region as ‘just right’ if it contained within ±10 percentiles of the median number of single nucleotide polymorphisms (SNPs) appearing over all regions of our genome wide association study. These regions were then sorted by the number of SNPs appearing within the corresponding regions in a separate genotyping chip study. This allowed us to maximize the number of sites at which to check consensus between a run of our quality control pipeline and the SNP chip.

Goldilocks was originally designed to return regions that matched these explicit criteria across multiple samples. The package has since been made more flexible and can be used to find regions of interest based on any arbitrary criteria such as GC-content, density of target motifs, pre-defined metrics and uncalled nucleotides (N’s).

## 2 Features

### 2.1 Customizable and interchangeable strategies

Criteria defining the metric for ‘interesting’ are held by Goldilocks in a user-selected strategy. Strategies are responsible for annotating regions of sequence data as interesting (or not) and quantifying the result as a summary statistic that can be used later for ranking.

Whilst Goldilocks includes a number of simple strategies for counting nucleotides, motifs, consensus to a reference and GC-content, the package offers a simple framework that allows one to customize or create strategies of their own (for an example refer to the supplement). Strategies implement the same interface and are thus all interchangeable, replacing the need for multiple stand-alone tools and data processing steps.

### 2.2 Simple sorting and flexible filtering

Goldilocks provides a simple but powerful query function capable of filtering and sorting regions by their absolute or percentile distance from the result of a mathematical operation over the whole set of regions, such as *max*, *min*, *mean*, *median* or proximity to an arbitrary target value. Candidates can be excluded based on the start or end position of the region, chromosome (or contig) and simple expressions on the strategy values themselves. Queries can be chained, allowing users to build up more complex queries to find regions with more specific criteria of interest.

### 2.3 Plotting and exporting

Goldilocks provides several functions for extracting collected region metadata and the results of queries on that data. Sequences that lie on regions identified as interesting can be exported as FASTA, regions themselves can be output as BED. The package can output publication-ready graphs via matplotlib, or region metadata as a ‘melted’ dataframe for easy plotting with external packages such as R’s ggplot2 (Wickham, 2009). Data may also be exported in an arbitrarily delimited format for import into virtually any other tool, including Circos (Krzywinski *et al.*, 2009). For examples, please refer to the supplement.

### 2.4 Multiprocessed census and efficient input

The census step can be executed over a user specified number of processes to improve performance on both desktops and servers. Sequence FASTA files are not read into physical RAM but instead mapped to virtual memory and accessed via indices stored in their corresponding index, avoiding expensive input reading operations.

On a Core i5 laptop using four processes, Goldilocks can calculate GC-content over a whole human genome (*hs37d5*) with a window size of 100 kbp (and overlap of 50 kbp) in 3 min while using less than 2 GB of memory. On the same machine Goldilocks could simultaneously take a census of 10 short nucleotide motifs with regular expressions and overlaps in 15 min or 50 simple motifs in just over 5 min and using less than 3 GB of memory.

## 3 Discussion

The flexibility of Goldilocks lends itself for use in many different scenarios, for example:

- **Summarizing** **genomes**Tabulate or plot variation in some statistic such as GC-content across one or more genomes for inspection.
- **Exploring** **extremes**Find regions demonstrating unusual extremes with respect to the rest of the sequence data, such as high numbers of repeating subsequences or low GC-content.
- **Identifying** **shifts**Detect shifts in variation in some statistic along a sequence, such as identification of chimeric activity within contigs.
- **Discovering** **outliers**Detect regions of sequences with properties indicative of data quality issues.
- **Seeding** **sequences**Locate subsequences to use as appropriate seeds for execution of other tools and algorithms.
- **Extracting** **subsets**From a large set of regions of sequence, extract a subset which meets some desired arbitrary criteria for further analysis.

Alternative counting mechanisms, such as those available in Galaxy (Giardine *et al.*, 2005) through EMBOSS (Rice *et al.*, 2000) or various statistics tools in the Galaxy Toolshed, can accomplish similar functionality. However, as different tools require specific input formats and produce a variety of output formats, additional data handling steps are required as part of any pipeline, making it difficult to customize or swap counting plugins.

Goldilocks is not intended to replace already existing dedicated software for counting tasks that require specific optimized data structures such as khmer (Crusoe *et al.*, 2014) for counting k-mers, but instead provides a general suite of swappable counters.

Genome browsers such as the UCSC Genome Browser (Kent *et al.*, 2002) allow the user to view a variety of tracks displaying the locations of different genomic properties. Interpreting large amounts of sequencing data by eye in such browsers can be difficult. Alternative browsers that allow zoomed browsing of interesting regions, such as the LayerCake (Correll *et al.*, 2015) visualization tool, attempt to ameliorate this problem. Goldilocks will automatically find regions of interest, suitable for further browsing or plotting if required.

Epiviz and Epivizr (Chelaru *et al.*, 2014) combine both statistical analysis and visualization, connecting a web-based browsing environment to an R/Bioconductor-based calculation environment. Users interactively add more tracks or plots to further explore the areas surrounding the regions of interest in the genome. Their platform is comprehensive and provides a far larger scope for analysis than Goldilocks. We see Goldilocks as a lightweight Python solution for straightforward queries, and a useful addition to existing bioinformatics pipelines.

Goldilocks has minimal dependencies: the core requires just NumPy (Oliphant, 2007) and matplotlib (Hunter, 2007) to enable plotting. We provide a command line tool that offers access to the base functionality of the package to users without having to write a script of their own (for example usage see the supplement). Goldilocks is packaged with a testing suite, results and coverage of which are available online along with documentation. Source code is publicly available under an open source licence for review and community contribution.

## Supplementary Material

Supplementary Data

## References

[btw116-B1] Chelaru F. (2014) Epiviz: interactive visual analytics for functional genomics data. Nat. Methods, 11, 938–940. 25086505 10.1038/nmeth.3038 PMC4149593

[btw116-B2] Correll M. (2015) LayerCake: a tool for the visual comparison of viral deep sequencing data. Bioinformatics, 31, 3522–3528. 26153515 10.1093/bioinformatics/btv407 PMC4626748

[btw116-B3] Crusoe M.R (2015). The khmer software package: enabling efficient nucleotide sequence analysis. F1000Research **4**, 900. 10.12688/f1000research.6924.1 PMC4608353 26535114

[btw116-B4] Giardine B. (2005) Galaxy: a platform for interactive large-scale genome analysis. Genome Res., 15, 1451–1455. 16169926 10.1101/gr.4086505 PMC1240089

[btw116-B5] Hunter J.D. (2007) Matplotlib: a 2d graphics environment. Comput. Sci. Eng., 9, 90–95.

[btw116-B6] Kent W.J. (2002) The human genome browser at UCSC. Genome Res., 12, 996–1006. 12045153 10.1101/gr.229102 PMC186604

[btw116-B7] Krzywinski M.I. (2009) Circos: an information aesthetic for comparative genomics. Genome Res., 19, 1639–1645. 19541911 10.1101/gr.092759.109 PMC2752132

[btw116-B8] Oliphant T.E. (2007) Python for scientific computing. Comput. Sci. Eng., 9, 10–20.

[btw116-B9] Rice P. (2000) EMBOSS: The European Molecular Biology Open Software Suite. Trends Genet., 16, 276–277. 10827456 10.1016/s0168-9525(00)02024-2

[btw116-B10] Wickham H. (2009) ggplot2: Elegant Graphics for Data Analysis. Springer, New York.

