# Supplementary material for: Goldilocks: a tool for identifying genomic regions that are ‘just right’
Source: Bioinformatics. 2016 Mar 7;32(13):2047–9. doi: 10.1093/bioinformatics/btw116 (PMC4920124; doi:10.1093/bioinformatics/btw116)
Supplement: Supplementary Data [file supp_32_13_2047__index.html]

Goldilocks: a tool for identifying genomic regions that are ‘just right’ — Goldilocks: a tool for identifying genomic regions that are ‘just right’ — Supplementary Data 

# Goldilocks: a tool for identifying genomic regions that are ‘just right’

## Supplementary Data

files

- Supplementary Data - pdf file
