# Supplementary material for: Goldilocks: a tool for identifying genomic regions that are ‘just right’
Source: Bioinformatics. 2016 Mar 7;32(13):2047–9. doi: 10.1093/bioinformatics/btw116 (PMC4920124; doi:10.1093/bioinformatics/btw116)
Supplement: Supplementary Data [file supp_btw116_BIOINF-2015-1318_SUPPLEMENT.pdf]

Goldilocks: A tool for identifying genomic regions that are "just right"

Supplement: Usage and plotting examples

Samuel M. Nicholls, Amanda Clare, Joshua C. Randall
msn@aber.ac.uk — https://github.com/SamStudio8/goldilocks

This document is provided as a supplement to our paper as an introductory guide to Goldilocks with examples of using the package to conduct a census and a demonstration of the formatted output and plotting functionality. Examples were tested at the time of print but Goldilocks is open-source software under continuous development and future versions may feature a different API. For more background, examples and the latest documentation refer to the canonical documentation hosted at https://goldilocks.readthedocs.org/en/latest/.

Contents

1 Installation **2**

2 Command Line Usage Example **2**
2.1 Inspect Strategies . . . 2
2.2 Nucleotide Counting . . . 3

3 Package Import Usage and Output Format Examples **4**
3.1 Nucleotide Counting . . . 4
3.2 Our Quality Control Study . . . 5
3.2.1 Tabulated Output . . . 6
3.2.2 BED Output . . . 6
3.2.3 Melted Output . . . 7
3.2.4 Circos Output . . . 7

4 Custom Census Rules **8**
4.1 A Simple ORF Finder . . . 8
4.1.1 Code Sample . . . 8
4.1.2 Implementation Description . . . 9

5 Plotting Examples **10**
5.1 Scatter Graphs . . . 10
5.1.1 Simple Plot . . . 10
5.2 Line Graphs . . . 11
5.2.1 Plot multiple contigs or chromosomes from one sample . . . 11
5.2.2 Plot a contig or chromosome from multiple samples . . . 12
5.3 Histograms . . . 13
5.3.1 Simple profile (binning) plot . . . 13
5.3.2 Simpler profile (binning) plot . . . 14
5.3.3 Proportional bin plot . . . 14
5.3.4 Bin multiple contigs or chromosomes from one sample . . . 15
5.3.5 Bin a contig or chromosome from multiple samples . . . 16
5.4 Advanced . . . 17
5.4.1 Plot data from multiple counting tracks from one sample's chromosomes . . . 17
5.4.2 Plot data from multiple counting tracks for one chromosome across many samples . . . 18
5.5 Integration with external plotting tools . . . 19
5.5.1 ggplot2 . . . 19
5.5.2 Circos . . . 20

1 Installation

Goldilocks is available from the Python package index. The latest version can be installed with `pip`.

```
> pip install goldilocks
```

The development version can be retrieved from the repository https://github.com/SamStudio8/goldilocks. Bug reports and feature requests may be submitted to the repository's issue page or the corresponding author.

2 Command Line Usage Example

2.1 Inspect Strategies

Although Goldilocks is a Python package designed primarily to be imported into user scripts, the package features a lightweight command line interface that exposes its basic functionality. Goldilocks is packaged with several built-in strategies that will cover the majority of basic counting tasks. To see the strategies and output formats that are available, execute `goldilocks list`:

```
> goldilocks list
Available Strategies
  * gc (GCRatioStrategy)
      Calculate GC ratio over regions
  * motif (MotifCounterStrategy)
      Count occurrences of one or more nucleotide motifs
  * nuc (NucleotideCounterStrategy)
      Count occurrences of one or more individual nucleotides
  * ref (ReferenceConsensusStrategy)
      Calculate (dis)similarity to a given reference

Available Output Formats
  * bed
      Browser Extensible Data containing region positions only
  * circos
      Data compatible with the circos plotting tool
  * melt
      Data compatible with tools expecting a melted dataframe, such as ggplot2
  * table
      Simple tabulated output
```

2.2 Nucleotide Counting

Consider the problem of finding regions of a genome that contain the most uncalled nucleotides (N's). As described in our paper, Goldilocks conducts a census of regions by sliding a window of a user-defined length and overlap over input sequences and applying a desired strategy.

In this scenario we want to apply the nucleotide counting strategy, referred to as `nuc` by the command line interface. A strategy may count multiple interesting targets simultaneously. For example the nucleotide counting strategy may count the presence of multiple bases in one census; Goldilocks refers to these multiple interesting targets as '*tracks*'. We specify one track, to count the number of appearances of the nucleotide N. Results should be sorted by `max` (*i.e.* in descending order). We will arbitrarily select the region length to be 100,000 base pairs and the stride (overlap) to be 50,000 base pairs. The example below demonstrates how to provide these options to the Goldilocks command line interface and process the sequences stored in files `sample0.fa.fai` to `sampleN.fa.fai`.

```
> goldilocks nuc \                # use the NucleotideCounter strategy
             max \                # sort the censused regions by maximum value
             --tracks N \         # count the N bases only
             -l 100000 \          # length of the census window
             -s 50000 \           # overlap of the census window
             -@ 4 \               # permit multi-threaded census with 4 threads
             sample0.fa.fai \     # input FASTA index(es)
              ...
             sampleN.fa.fai \
```

By default Goldilocks outputs census results in a tab-delimited format to `stdout`. Each row in the table below represents a censused region on a chromosome, with a 1-indexed start and end base. A column for each sample (input file) and track (in this case, just the nucleotide 'N') combination is appended to the table. For the purpose of our example, for each region, the number of N nucleotides seen on `sample0.fa.fai` will appear under `0_N` and so on. Columns continue up to `N_N`, the number of N bases seen on some `sampleN.fa.fai`, for each region.

As we've specified `max`, the region rows will be sorted by the total number of N bases seen on that region across all of the samples 0..N. For more complex sorting, users will need to use the package in their own scripts instead.

chr	pos_start	pos_end	0_N	1_N	...	N_N
1	100001	150000	25000	12500	...	37500
...	...	...	...	...	...	...
1	1	50000	0	25000	...	1500

Goldilocks supports additional output formats, as listed by the command `goldilocks list`, that are introduced in Section 3.2. These can be selected by specifying the `-f` option: *e.g.* `-f circos`. However, for more information and examples of currently available output formats, refer to the package's online documentation.

3 Package Import Usage and Output Format Examples

Usage of Goldilocks is not limited to the command line, in fact one may access its more advanced features by importing the package to their own scripts. Note that Goldilocks is distributed under the MIT license, allowing end users to use the software in their own programs without restriction.

3.1 Nucleotide Counting

Let's repeat the introductory command line example from earlier by using the package in a Python script. Let's consider the code sample below, we begin by importing the Goldilocks package, the desired strategy and a dictionary describing the sources of sequence data. Each key in this dictionary is the name of a *sample* (a loosely defined term) whose corresponding value is another dictionary that contains the single key `file` with value contains the path to the FASTA index file for that particular sample.

The census options are then defined as arguments to the `Goldilocks` object constructor. The desired strategy is the first positional parameter and is instantiated with a Python list containing the counting tracks of interest; in this case, the single nucleotide N. The `sequence_data` dictionary is the second positional parameter.

The remaining keyword arguments may appear in any order and simply encode the desired parameters of the census in a similar fashion to the command line example. `length` and `stride` set the length and overlap of the census region, respectively, the number of processes may be set with the `processes` keyword. `is_faidx` is a special boolean parameter that ensures the `sequence_data` dictionary is treated as containing paths to FASTA index files, as opposed to actual sequence data.

Finally, after the census is completed, one can use the `query` function to analyse the results. In this example we sort regions by the `max` of total 'N' nucleotides seen on each region across all the input samples (as before).

```
# Import Goldilocks itself
from goldilocks import Goldilocks

# Import the desired strategy from goldilocks.strategies
# Refer to documentation or execute "goldilocks list" for available strategies
from goldilocks.strategies import NucleotideCounterStrategy

# Sequence data, keys are sample/group names
# Values are dictionaries with single key file and a path to particular FASTA index as value
sequence_data = {
    "sample0": {"file": "/path/to/sample0.fa.fai"},
    ...
    "sampleN": {"file": "/path/to/sampleN.fa.fai"},
}

# Construct the Goldilocks census
g = Goldilocks(
        NucleotideCounterStrategy(["N"]),  # Instantiate strategy with track(s) of interest
        sequence_data,                     # Provide sequence data dictionary
        length="100K", stride="50K",       # Set the length and overlap of the census window
        is_faidx=True,                     # Flag to treat data as FASTA index files
        processes=4                        # Permit 4 threads during census
)

# Query the census, sort regions by "max" number of N's, print a simple comma-delimited
#  table of the total N's seen on each region, and output a FASTA of regions with
#  the highest number of occurrences over all samples
g.query("max").export_meta(sep=",", group="total")
g.export_fasta()
```

3.2 Our Quality Control Study

Goldilocks is not limited to simple queries, in fact queries can be chained together to identify regions that satisfy a complex set of criteria. As briefly described in our paper, Goldilocks was originally written to locate regions of the human genome that expressed a “representative” degree of variation (that is, the density of single nucleotide polymorphisms (SNP)) across data in a genome wide association study, whilst maximising the number of SNP sites that could be compared between an individual’s whole genome sequence and run of a genotyping chip.

The chromosome-base position pairs analysed by both the whole genome study (the `gwas` group) and the genotype chip study (`ichip`) were available in a tab-delimited file (extracted from the `CHR`, `POS` columns of the VCF). Goldilocks is capable of automatically parsing this “position format” and thus the sequence data dictionary can be specified in a similar manner to those for FASTA index files.

The Goldilocks object is constructed in the same way, despite the use of a different strategy. Here we initialise the `PositionCounterStrategy`: a special strategy that reads lists of positions rather than actual sequence data. This particular strategy does not require any tracks to be set.

The region length is 1Mbp with a 500Kbp overlap. Note the use of the `is_pos_file` flag rather than `is_faidx`, as the inputs are tab-delimited position files, not FASTA index files. It is anticipated in future releases that Goldilocks will be capable of detecting whether or not users are providing files or raw sequence data to remove the requirement of raising a flag to declare the input file type.

It is also possible to set the number of simultaneous processes to be spawned during a census with `processes`.

```
from goldilocks import Goldilocks
from goldilocks.strategies import PositionCounterStrategy

sequence_data = {
    "gwas": {"file": "/encrypt/ngsqc/vcf/cd-seq.vcf.q"},
    "ichip": {"file": "/encrypt/ngsqc/vcf/cd-ichip.vcf.q"},
}

g = Goldilocks(PositionCounterStrategy(), sequence_data,
        length="1M", stride="500K", is_pos_file=True, processes=8)

g.query("median", percentile_distance=20, group="gwas", exclusions={"chr": [6]})
g.query("max", percentile_distance=5, group="ichip", limit=25)
```

Once the census is complete, we sort the number of variants seen on the `GWAS` *group* at each region by their distance from the `median` of variants seen on the GWAS over all regions. We filter out any regions that do not appear within ±10 percentiles of the median with `percentile_difference`. We exclude any region from chromosome 6 with an `exclusions` dictionary (we wanted to avoid the major histocompatibility complex). The syntax for `exclusions` and other keywords are described in more detail in the online documentation.

To enhance our study and retrieve the subset of these regions that will best maximise the number of locations. We can compare results between the GWAS and separate chip studies, we conduct another `query` on the Goldilocks object. This time, sorting by the maximum number of variants seen only on the `ichip` *group*. We filter out any regions that are not in the top five percentiles and limit the maximum number of results to 25.

We can then view the results with `export_meta`. The following subsections provide an example of the output formats available at the time of publishing. For the latest available formats, execute `goldilocks list` or consult the online documentation.

3.2.1 Tabulated Output

This is the default option if no `fmt` argument is provided to `export_meta`. The format itself was described in our earlier command line usage example.

```
g.export_meta()
```

chr	pos_start	pos_end	gwas_count	ichip_count
2	102000001	103000000	173.0	1928.0
3	46000001	47000000	173.0	1540.0
2	100000001	101000000	179.0	1397.0
...	...	...	...	...
3	159000001	160000000	158.0	724.0
2	234000001	235000000	179.0	721.0
2	233500001	234500000	149.0	715.0

3.2.2 BED Output

One may just be interested in the regions and the specific values of the counts themselves are inconsequential. Goldilocks can write just the metadata for the locations of regions in the widely accepted `BED` format. Note that positions are 0-indexed and only the first three columns required by the specification are populated.

```
g.export_meta(fmt="bed")
```

chrom	chromStart	chromEnd
2	102000000	102999999
3	46000000	46999999
2	100000000	100999999
...	...	...
3	159000000	159999999
2	234000000	234999999
2	233500000	234499999

3.2.3 Melted Output

Often, it is useful to analyse results when they are in a "melted" format: that is, a region will feature census data across many rows instead of many columns. This is especially popular with users of the R language.

```
g.export_meta(fmt="melt")
```

```
region  region_id  group_track   group  track  chr  chr_i  value
0       701        gwas-count    gwas   count  2    204    173.0
0       701        ichip-count   ichip  count  2    204    1928.0
1       1074       gwas-count    gwas   count  3    92     173.0
1       1074       ichip-count   ichip  count  3    92     1540.0
2       697        gwas-count    gwas   count  2    200    179.0
2       697        ichip-count   ichip  count  2    200    1397.0
...     ...        ...           ...    ...    ...  ...    ...
22      1300       gwas-count    gwas   count  3    318    158.0
22      1300       ichip-count   ichip  count  3    318    724.0
23      965        gwas-count    gwas   count  2    468    179.0
23      965        ichip-count   ichip  count  2    468    721.0
24      964        gwas-count    gwas   count  2    467    149.0
24      964        ichip-count   ichip  count  2    467    715.0
```

The region field represents the sequentially numbered i'th region in the output itself, whereas region_id refers to the region's actual index out of all regions (these are sequentially ordered along the input genome). The group-track, group and track describe the sample and counting track for the melted region. chr is the number or name of the chromosome or contig. chr_i is the index of the current region on chr, this can be useful as an x-axis when plotting regions along particular chromosomes.

3.2.4 Circos Output

It is also possible to output metadata immediately compatible for plotting with the popular circos tool.

```
g.export_meta(fmt="circos", chr_prefix="hs", value_bool=True, header=False, group="total")
```

```
hs2 102000001 103000000 1
hs3 46000001 47000000 1
hs2 100000001 101000000 1
... ...       ...       ...
hs3 159000001 160000000 1
hs2 234000001 235000000 1
hs2 233500001 234500000 1
```

Several keyword arguments are available for export_meta that we have taken advantage of here. As the karyotype data file we used to generate plots with circos had chromosome sequences named hs1..hsY, we added the chr_prefix keyword to automatically prepend the sample chromosome's with hs to match without manual editing later. Setting value_bool replaces the final value column with 1 for any region with a value greater than 0, and 0 otherwise; this comes in useful for tracks with circos that just require a boolean value. To remove the header line we set header to False. The group parameter is set to total (as opposed to either the gwas or ichip sample groups). The total sample group is created automatically and holds the sum of census values seen on a given region across all samples. This is set to prevent export_meta printing rows for both groups.

4 Custom Census Rules

One of the major features of Goldilocks is its extensibility. Strategies are both easily customisable and interchangeable, as they all share a common interface. This interface also provides a platform for users with some knowledge of Python to construct their own custom census rules. One such example follows below:

4.1 A Simple ORF Finder

4.1.1 Code Sample

```python
# Import Goldilocks and the BaseStrategy class
from goldilocks import Goldilocks
from goldilocks.strategies import BaseStrategy

# Define a new class for your custom strategy that inherits from BaseStrategy
class MyCustomSimpleORFCounterStrategy(BaseStrategy):

    # Initialising function boilerplate, required to set-up some properties of the census
    def __init__(self, tracks=None, min_codons=1):
        # Initialise the custom class with super
        super(MyCustomSimpleORFCounterStrategy, self).__init__(
                tracks=range(0,3),                     # Use range to specify a counter for
                                                       #  each of the three possible forward
                                                       #  reading frames in which to search
                                                       #  to search for open reading frames
                label="Forward Open Reading Frames"    # Y-Axis Plot Label
        )
        self.MIN_CODONS = min_codons

    # This function defines the actual behaviour of a census for a given region
    #  of sequence and the current counting track (one of three reading frames)
    def census(self, sequence, track_frame, **kwargs):
        STARTS = ["ATG"]
        STOPS = ["TAA", "TGA", "TAG"]
        CODON_SIZE = 3

        # Split input sequence into codons. Open a frame if a START is found
        #  and increment the ORF counter if a STOP is encountered afterward
        orfs = orf_open = 0
        for i in xrange(track_frame, len(sequence), CODON_SIZE):
            codon = sequence[i:i+CODON_SIZE].upper()
            if codon in STARTS and orf_open == 0:
                orf_open = 1
            elif codon in STOPS and orf_open > 0:
                if orf_open > self.MIN_CODONS:
                    orfs += 1
                orf_open = 0
            elif orf_open > 0:
                orf_open += 1
        return orfs

# Organise and execute the census
sequence_data = { "hs37d5": {"file": "/store/ref/hs37d5.1-3.fa.fai"} }
g = Goldilocks(MyCustomSimpleORFCounterStrategy(min_codons=30), sequence_data,
        length="1M", stride="1M", is_faidx=True, processes=4)
```

4.1.2 Implementation Description

Strategies are defined as Python classes, inheriting from the `BaseStrategy` class found in the `goldilocks.strategies` subpackage. The class requires just two function definitions to be compliant with the shared interface; `__init__`: the class initializer that takes care of the setup of the strategy's internals via the `BaseStrategy` parent class, and `census`: the function actually responsible for the behaviour of the strategy itself.

The example presented is a very simple open reading frame counter. It searches the three forward frames for start codons that are then followed by one of the three stop codons. The "tracks" in this example are the three possible frames. Note on line 9 that our `__init__` provides a default argument for `tracks` of `None`. Thus this particular strategy does not need the `tracks` argument. Instead, the track list is provided by the strategy itself, and passed to the `BaseStrategy` `__init__` (line 12), forcing tracks to be the list [0, 1, 2]. The elements of this list are used as an integer offset from which to begin splitting input DNA sequences when conducting the census later, which is why on this occasion we don't want to allow the user to specify their own tracks. Other strategies, such as the included `NucleotideCounterStrategy` just pass the `tracks` argument from the user through to the super `__init__`.

For a given array of `sequence` data and a frame offset (`track_frame`), the `census` function splits the sequence into nucleotide triplets from the offset and searches for open reading frames. A subsequence is considered an ORF by this strategy if the ATG START codon is encountered and later followed by any STOP codon.

Our example finishes with the familiar specification of the location of input sequence data and the construction of the census itself. Here we specify a census of all 1Mbp regions with no overlap (that is, the stride is equal to the size of the regions) and instantiate our new `MyCustomSimpleORFCounterStrategy` with a keyword requiring valid ORFs to be at least 30 codons in length (excluding start and stop).

Every strategy's `census` function is expected to return a numerical result that can be used to rank and sort regions, in this scenario, `census` returns the number of ORFs found.

Note also, strategies may specify any number of keyword arguments that are not found in the `BaseStrategy`. In our example, `min_codons` can be set by a user to specify how many codons must lie between an opening and closing codon to be counted as an open reading frame. We store this value as a member of the strategy object on line 18 and use it on line 35 to ensure the `orfs` counter is only incremented when the length of the current open reading frame has exceeded the provided threshold. One could store any number of configurable parameters inside of the strategy class in this fashion. This framework allows one to increase the complexity of strategies while still providing a friendly and interchangeable interface for end users.

5 Plotting Examples

5.1 Scatter Graphs

5.1.1 Simple Plot

After executing a census one can use the `plot` function to create a scatter graph of results. The `x` axis is the location along the genome (with ordered chromosomes or contigs appearing sequentially) and the `y` axis is the value of the censused region according to the strategy used. The example below plots GC content ratio across the first three chromosomes of the `hs37d5` reference sequence, with a window size of 100,000 and a step or overlap of 50,000. Note that the plot title may be specified with the `title` keyword argument.

```
from goldilocks import Goldilocks
from goldilocks.strategies import GCRatioStrategy

sequence_data = {
    "my_sequence": {"file": "/store/ref/hs37d5.1-3.fa.fai"},
}
g = Goldilocks(GCRatioStrategy(), sequence_data, length="100K", stride="50K", is_faidx=True)
g.plot(title="GC Content over hs37d5 Chr1-3")
```

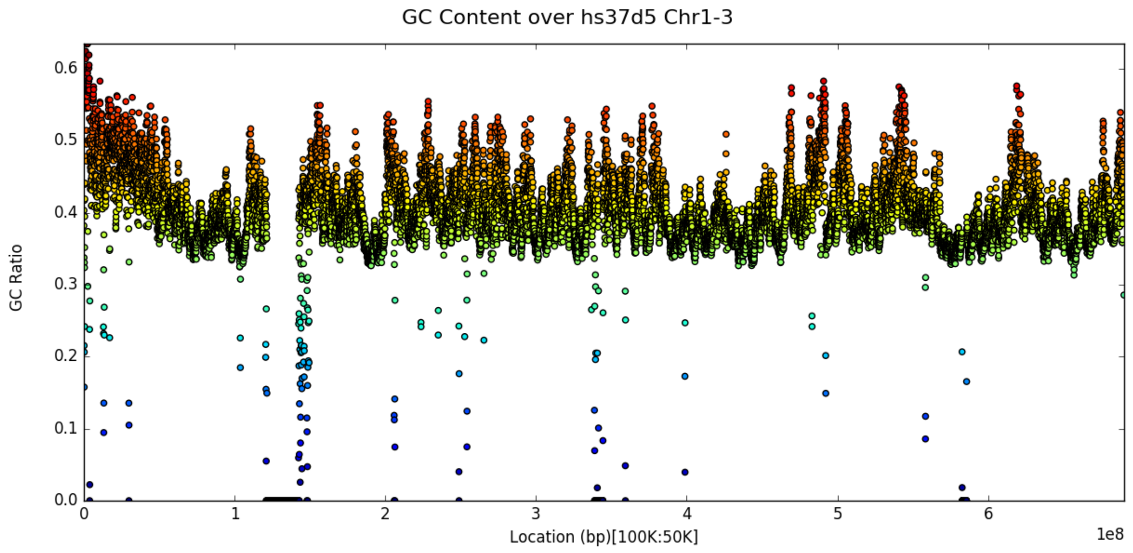

5.2 Line Graphs

5.2.1 Plot multiple contigs or chromosomes from one sample

For long genomes or a census with a small window size, simple plots as shown in the previous section can appear too crowded and thus difficult to extract information from. One can instead plot, for a given input sample, a panel of census region data, by chromosome by specifying the name of the sample as the first parameter to the `plot` function as per the example below:

```
from goldilocks import Goldilocks
from goldilocks.strategies import GCRatioStrategy

sequence_data = {
    "hs37d5": {"file": "/store/ref/hs37d5.1-3.fa.fai"},
    "GRCh38": {"file": "/store/ref/Homo_sapiens.GRCh38.dna.chromosome.1-3.fa.fai"},
}
g = Goldilocks(GCRatioStrategy(), sequence_data, length="1M", stride="250K", is_faidx=True)
g.plot("hs37d5", title="GC Content over hs37d5 Chr1-3")
```

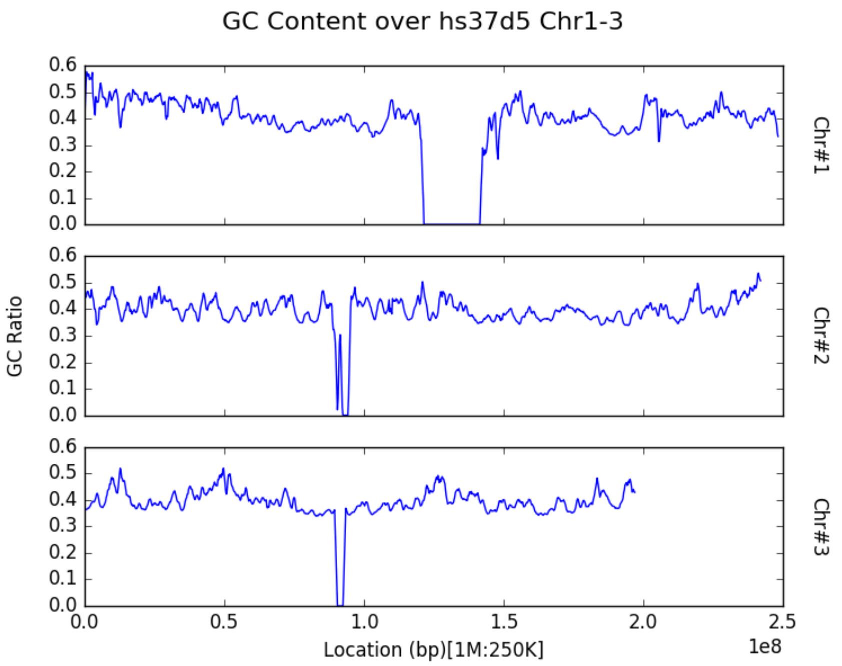

Note that both the x and y axes are shared between all panels to avoid the automatic creation of graphics with the potential to mislead readers on a first glance by not featuring the same axes ticks.

5.2.2 Plot a contig or chromosome from multiple samples

By default, data within the census is aggregated by region across all input samples (in the `sequence_data` dictionary) for the entire genome. However, one may be interested in comparisons across samples, rather than between chromosomes in a single sample. One can plot the census results for a specific contig or chromosome for each of the input samples, by specifying the `chrom` keyword argument to the `plot` function. Take note that the argument refers to the sequence that appears as the i'th contig of each of the input FASTA and not the actual name or identifier of the chromosome itself.

```
from goldilocks import Goldilocks
from goldilocks.strategies import GCRatioStrategy

sequence_data = {
    "hs37d5": {"file": "/store/ref/hs37d5.1.fa.fai"},
    "GRCh38": {"file": "/store/ref/Homo_sapiens.GRCh38.dna.chromosome.1.fa.fai"},
}
g = Goldilocks(GCRatioStrategy(), sequence_data, length="1M", stride="250K", is_faidx=True)
g.plot(chrom=1, title="GC Content over Chr1")
```

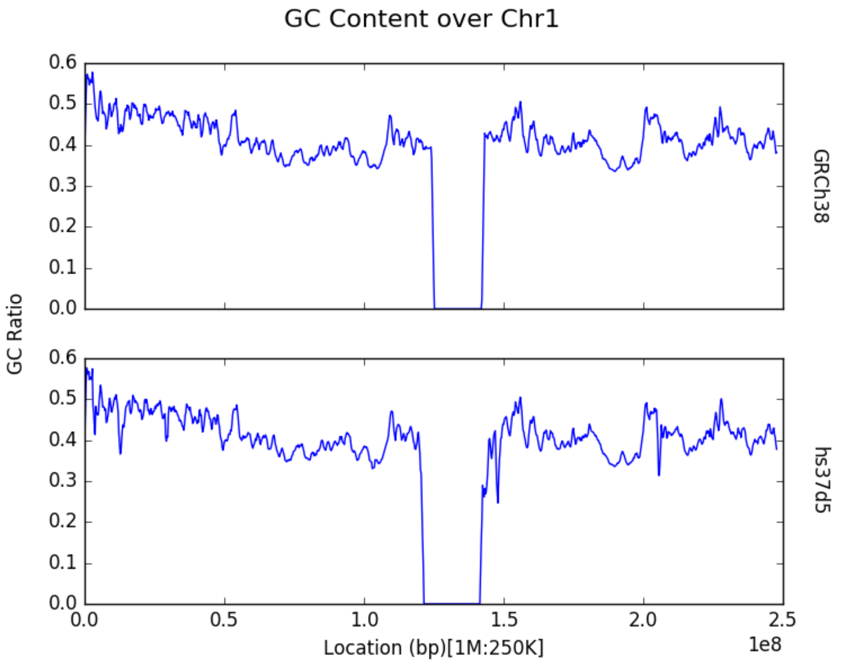

5.3 Histograms

5.3.1 Simple profile (binning) plot

Rather than inspection of individual data points, one may want to know how census data behaves as a whole. The `plot` function provides functionality to *profile* the results of a census through a histogram. Users can do this by providing a list of bins to the `bins` keyword argument of the `plot` function, following a census.

The example below shows the distribution of GC content ratio across the `hs37d5` reference sequence for all 100Kbp regions (and step of 50Kbp). The `x` axis is the bin and the `y` axis represents the number of censused regions that fell into a particular bin.

```
from goldilocks import Goldilocks
from goldilocks.strategies import GCRatioStrategy

sequence_data = {
    "my_sequence": {"file": "/store/ref/hs37d5.fa.fai"}
}

g = Goldilocks(GCRatioStrategy(), sequence_data,
        length="100K", stride="50K", is_faidx=True)

g.plot(bins=[0.0, 0.1, 0.2, 0.3, 0.4, 0.5, 0.6, 0.7, 0.8, 0.9, 1.0],
       title="GC Content Profile of hs37d5"
)
```

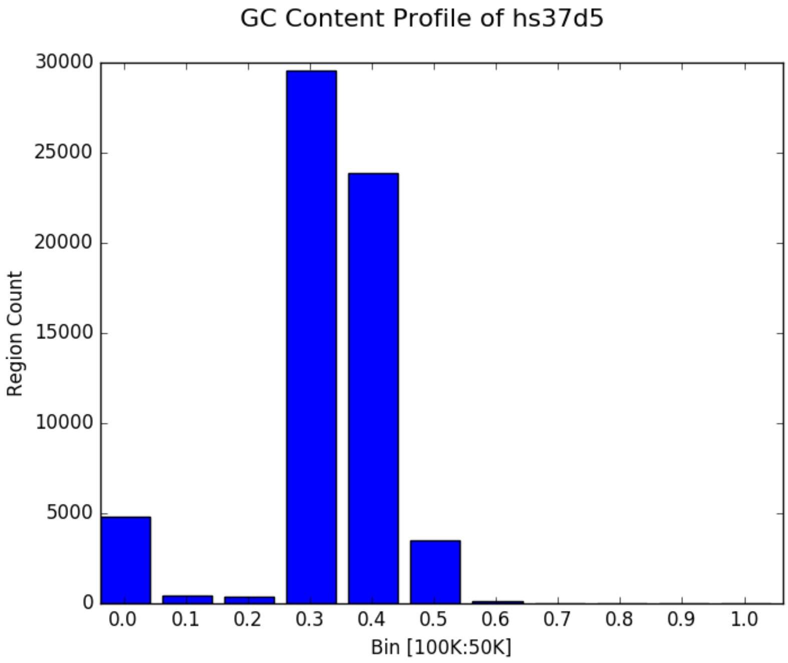

5.3.2 Simpler profile (binning) plot

It's trivial to select some sensible bins for the plotting of GC content as we know that the value for each region must fall between 0 and 1. However, many strategies will have an unknown minimum and maximum value and it can thus be difficult to select a suitable binning strategy without resorting to trial and error.

Thus the `plot` function permits a single integer to be provided to the `bins` keyword instead of a list. This will automatically create $N+1$ equally sized bins (reserving a special bin for 0.0) between 0 and the maximum observed value for the census. It is also possible to manually set the size of the largest bin with the `bin_max` keyword argument. The following example creates the same graph as the previous subsection, but without explicitly providing a list of bins.

```
from goldilocks import Goldilocks
from goldilocks.strategies import GCRatioStrategy

sequence_data = {
    "my_sequence": {"file": "/store/ref/hs37d5.fa.fai"},
}
g = Goldilocks(GCRatioStrategy(), sequence_data, length="100K", stride="50K", is_faidx=True)
g.plot(bins=10, bin_max=1.0, title="GC Content Profile of hs37d5")
```

5.3.3 Proportional bin plot

Often it can be useful to compare the size of bins in terms of their proportion rather than raw counts alone. This can be accomplished by specifying `prop=True` to `plot`. The `y` axis is now the percentage of all regions that were placed in a particular bin instead of the raw count.

```
from goldilocks import Goldilocks
from goldilocks.strategies import GCRatioStrategy

sequence_data = {
    "my_sequence": {"file": "/store/ref/hs37d5.fa.fai"}
}

g = Goldilocks(GCRatioStrategy(), sequence_data,
        length="100K", stride="50K", is_faidx=True)
g.plot(bins=10, bin_max=1.0, prop=True, title="GC Content Profile of hs37d5")
```

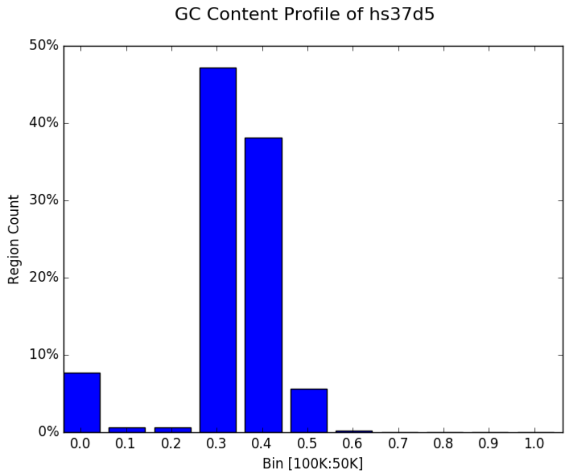

5.3.4 Bin multiple contigs or chromosomes from one sample

As demonstrated with the line plots earlier, one may also specify a sample name as the first parameter to `plot` to create a figure with each contig or chromosome's histogram on an individual panel.

```
from goldilocks import Goldilocks
from goldilocks.strategies import GCRatioStrategy

sequence_data = {
    "my_sequence": {"file": "/store/ref/hs37d5.1-3.fa.fai"}
}

g = Goldilocks(GCRatioStrategy(), sequence_data,
        length="100K", stride="50K", is_faidx=True)

g.plot("my_sequence",
       bins=10, bin_max=1.0, prop=True, title="GC Content Profiles of hs37d5 Chrs 1-3")
```

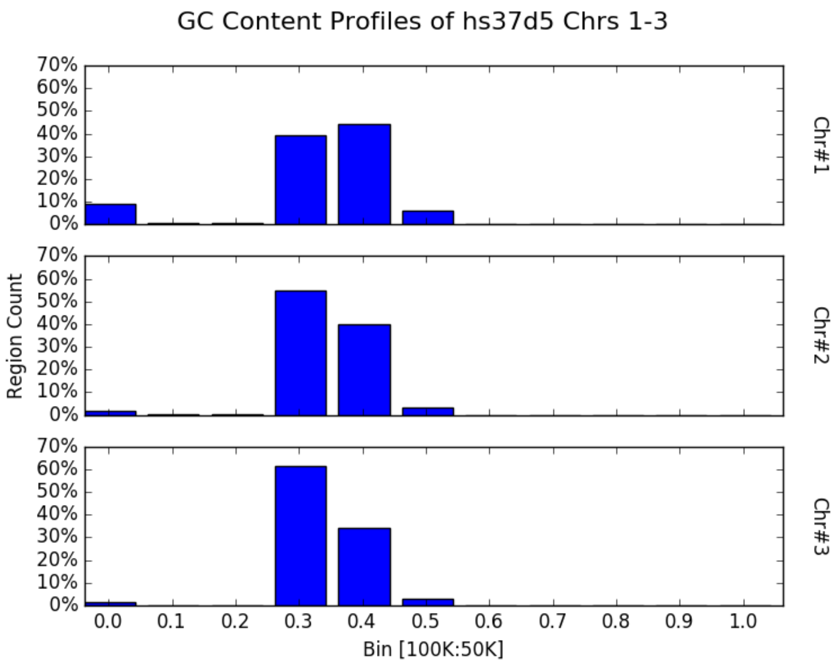

5.3.5 Bin a contig or chromosome from multiple samples

Similarly, one may want to profile a single contig or chromosome between each input group as previously demonstrated by the line graphs.

```
from goldilocks import Goldilocks
from goldilocks.strategies import GCRatioStrategy

sequence_data = {
    "hs37d5": {"file": "/store/ref/hs37d5.1.fa.fai"},
    "GRCh38": {"file": "/store/ref/Homo_sapiens.GRCh38.dna.chromosome.1.fa.fai"}
}

g = Goldilocks(GCRatioStrategy(), sequence_data,
        length="100K", stride="50K", is_faidx=True)
g.plot(chrom=1, bins=10, bin_max=1.0, prop=True, title="GC Content Profiles over Chr 1")
```

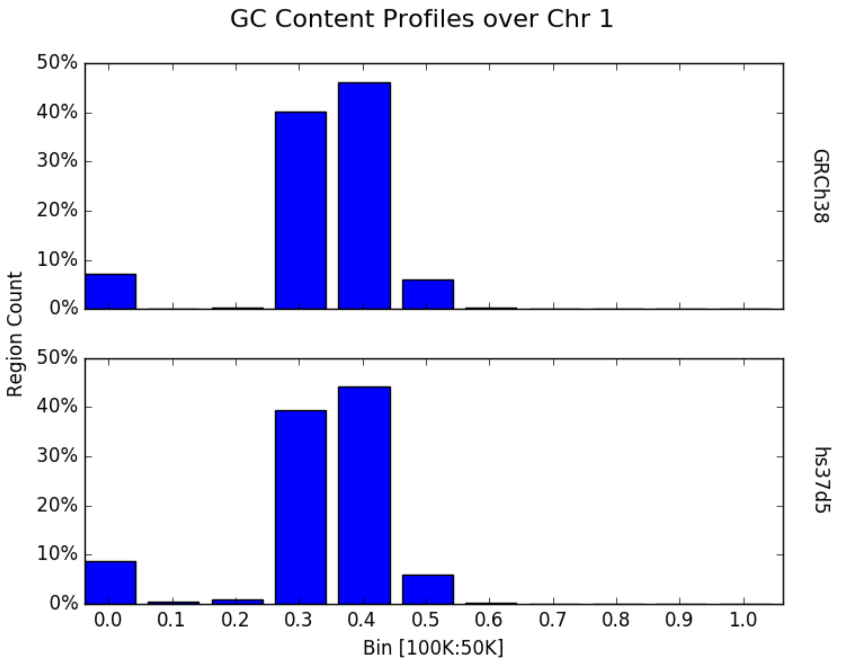

5.4 Advanced

5.4.1 Plot data from multiple counting tracks from one sample's chromosomes

The examples thus far have demonstrated plotting the results of a strategy responsible for counting one interesting property. But as demonstrated in Section 2.1, strategies are capable of counting multiple targets of interest simultaneously. Of course, one may wish to plot the results of all tracks rather than just the totals - especially for cases such as nucleotide counting where the sum of all counts will typically equal the size of the census region! The `plot` function accepts a list of track names to plot via the `tracks` keyword argument. Each counting track is then drawn on the same panel for the appropriate chromosome. A suitable legend is automatically placed at the top of the figure.

```
from goldilocks import Goldilocks
from goldilocks.strategies import NucleotideCounterStrategy

sequence_data = {
    "hs37d5": {"file": "/store/ref/hs37d5.1-3.fa.fai"},
}

g = Goldilocks(NucleotideCounterStrategy(["A", "C", "G", "T", "N"]), sequence_data,
        length="1M", stride="500K", is_faidx=True, processes=4)
g.plot(group="hs37d5", prop=True, tracks=["A", "C", "G", "T", "N"])
```

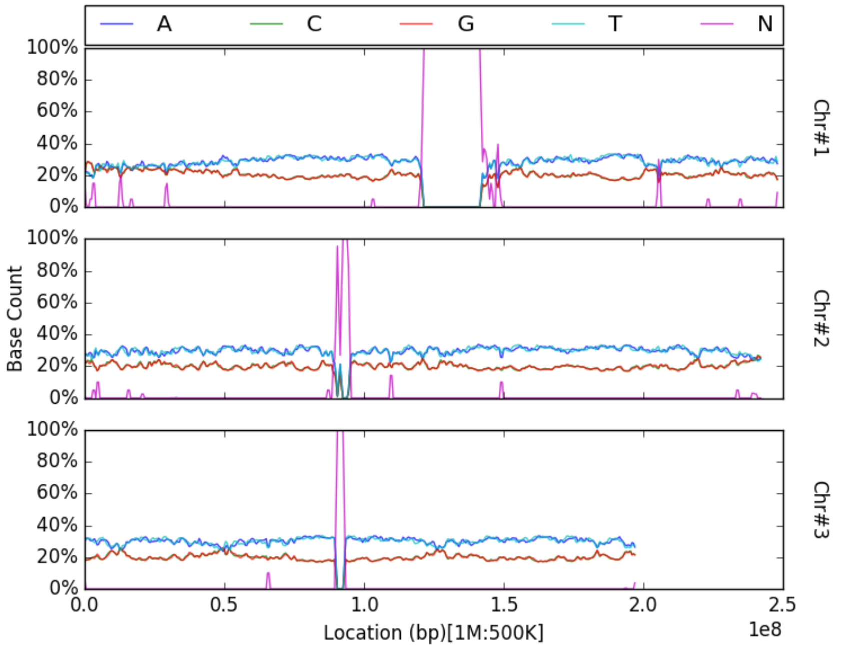

Note that `prop` is not a required argument, but can still be used with the `tracks` list to plot counts proportionally.

5.4.2 Plot data from multiple counting tracks for one chromosome across many samples

As seen in Section 5.2.1, one can use the `chrom` keyword argument for `plot` to create a figure featuring a panel per input sample, displaying census results for a particular chromosome. Similarly, this feature is supported when plotting multiple tracks with the `tracks` keyword.

```
from goldilocks import Goldilocks
from goldilocks.strategies import NucleotideCounterStrategy

sequence_data = {
    "hs37d5": {"file": "/store/ref/hs37d5.1.fa.fai"},
    "GRCh38": {"file": "/store/ref/Homo_sapiens.GRCh38.dna.chromosome.1.fa.fai"},
}

g = Goldilocks(NucleotideCounterStrategy(["A", "C", "G", "T", "N"]), sequence_data,
        length="1M", stride="500K", is_faidx=True, processes=4)
g.plot(chrom=1, prop=True, tracks=["A", "C", "G", "T", "N"])
```

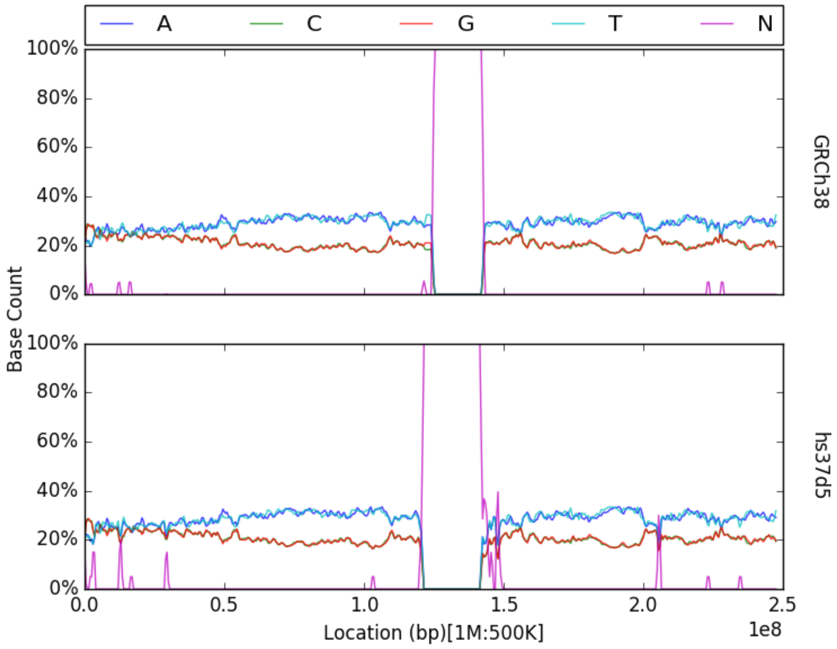

5.5 Integration with external plotting tools

5.5.1 ggplot2

Plotting packages such as `ggplot2` favour "melted" input as described in Section 3.2.2. The figure below was created using data from Goldilocks as part of our quality control study, the scatter plot compares the density of SNPs between the GWAS and SNP chip studies across the human genome.

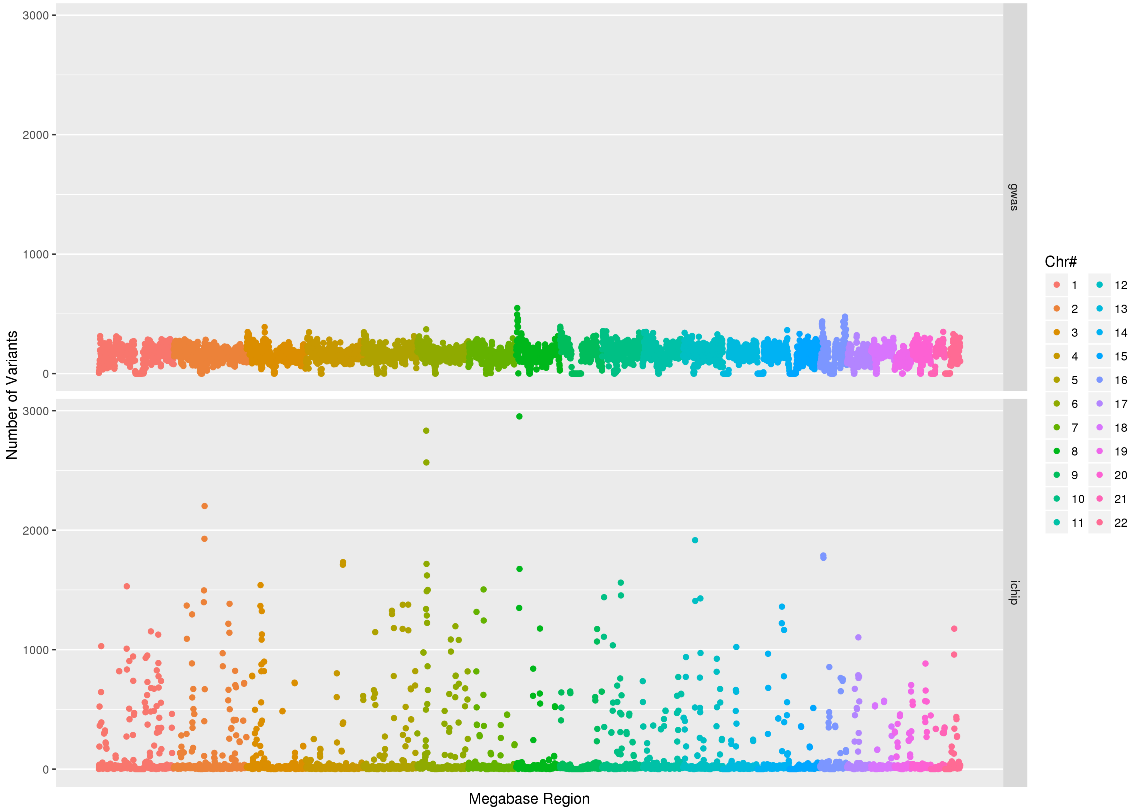

5.5.2 Circos

As demonstrated in Section 3.2.3, Goldilocks has an output format specifically designed to output information for use with the "popular and pretty" `circos` visualisation tool. Below is an example of a figure that can be generated from data gathered by Goldilocks. The figure visualises the selection of regions from our original quality control study. The Python script used to generate the data follows. The configuration for the plot itself has been excluded for the sake of brevity but can be downloaded from the online documentation.

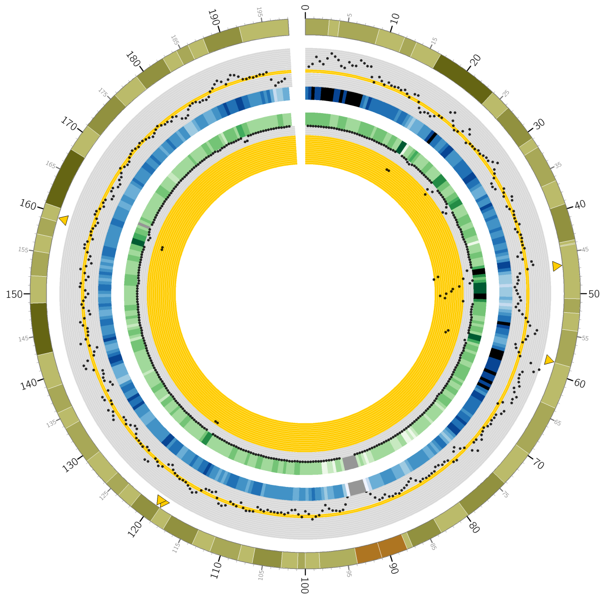

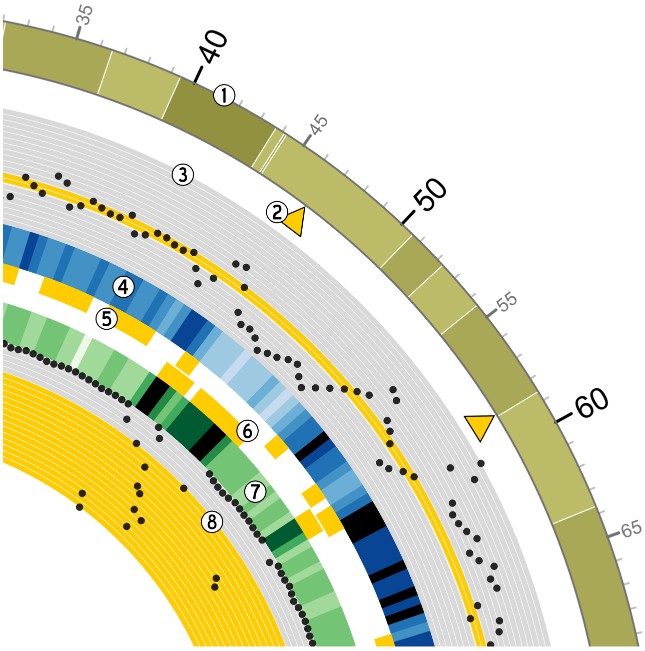

Sector of Goldilocks Circos Graph for Chr3 35:65Mbp
From outside inward: (1) Cytogenetic banding, (2) overall Goldilocks region indicator, (3) scatter plot and (4) heatmap of variants over GWAS samples, (5) GWAS and (6) SNP chip specific Goldilocks region indicators, (7) heatmap and (8) scatter plot of variants over SNP chip study. Scatter plots (3) and (8) are annotated with Goldilocks zones in gold, representing 10%iles around the median and top 5%iles respectively.
Note detection of two 1Mbp Goldilocks regions at 46-47Mbp and 58-59Mbp. The former was ultimately chosen as the "Goldilocks" region for our QC study.

```python
from goldilocks import Goldilocks
from goldilocks.strategies import PositionCounterStrategy

sequence_data = {
    "gwas": {"file": "/encrypt/ngsqc/vcf/cd-seq.vcf.q"},
    "ichip": {"file": "/encrypt/ngsqc/vcf/cd-ichip.vcf.q"},
}

g = Goldilocks(PositionCounterStrategy(), sequence_data,
        length="1M", stride="500K", is_pos_file=True)

# Query for regions that meet all criteria across both sample groups
# The output file goldilocks.circ is used to plot the yellow triangular indicators
g.query("median", percentile_distance=20, group="gwas", exclusions={"chr": [6]})
g.query("max", percentile_distance=5, group="ichip")
g.export_meta(fmt="circos", group="total", value_bool=True, chr_prefix="hs", to="goldilocks.circ")
```

```
# Reset the regions selected and saved by queries
g.reset_candidates()

# Export all region counts for both groups individually
# The -all.circ files are used to plot the scatter plots and heatmaps
g.export_meta(fmt="circos", group="gwas", chr_prefix="hs", to="gwas-all.circ")
g.export_meta(fmt="circos", group="ichip", chr_prefix="hs", to="ichip-all.circ")

# Export region counts for the groups where the criteria are met
# The -candidates.circ files are used to plot the yellow 'bricks' that
#   appear between the two middle heatmaps
g.query("median", percentile_distance=20, group="gwas")
g.export_meta(fmt="circos", group="gwas", to="gwas-candidates.circ")
g.reset_candidates()
g.query("max", percentile_distance=5, group="ichip")
g.export_meta(fmt="circos", group="ichip", to="ichip-candidates.circ")
g.reset_candidates()
```
